# Metastatic Invasive Lobular Carcinoma to Multiple Gastric Fundic Gland Polyps: A Rare Pattern of Gastric Involvement

**DOI:** 10.7759/cureus.90511

**Published:** 2025-08-19

**Authors:** Ahmed Jamshed, David Cartwright, Kevin Turner, Khalid Amin, Oyedele Adeyi, Byoung Uk Park

**Affiliations:** 1 Department of Laboratory Medicine and Pathology, University of Minnesota, Minneapolis, USA; 2 Department of Laboratory Medicine and Pathology, University of Alabama at Birmingham, Birmingham, USA

**Keywords:** fundic gland polyp, gastric metastasis of breast cancer, gastric polyps, invasive lobular carcinoma of the breast, late recurrence breast cancer

## Abstract

Breast cancer is one of the most common causes of cancer-related deaths among women. Here, we report the case of a 62-year old female with a history of invasive lobular carcinoma (ILC) of the breast, initially treated with neoadjuvant chemotherapy and mastectomy, who presented five years later with anemia. Endoscopic evaluation revealed over 50 gastric fundic gland polyps, with 8/10 sampled polyps showing metastatic involvement with ILC. Genetic testing showed no mutations in the *CTNNB1* or *APC* genes. This case raises the question of whether fundic gland polyps provide a unique microenvironment favorable for metastatic seeding. It also illustrates the importance of maintaining a high index of suspicion for disease recurrence or metastasis in the differential diagnosis of gastric findings, even years after the initial diagnosis and treatment of ILC.

## Introduction

Breast cancer remains the most diagnosed malignancy in women and is the second leading cause of cancer-related deaths among women globally [1,2]. In 2020 alone, breast cancer accounted for 11.7% of all new cancer diagnoses, with approximately 1 in 8 women projected to be diagnosed with invasive breast cancer, of which 1 in 43 are anticipated to die from the disease [1,2]. Invasive ductal carcinoma (IDC) and invasive lobular carcinoma (ILC) are the two predominant histologic subtypes of invasive breast cancer. While ILC is less common and constitutes approximately 14% of all malignant breast tumors [3,4] due to its diagnostic challenges and distinctive clinical behavior, it remains a significant clinical entity [5].

ILC is unique with its morphology, clinicopathological features, and metastatic behavior, and is characterized by loss of E-cadherin expression, a transmembrane protein responsible for epithelial cell-cell adhesion. The absence of E-cadherin, driven by genetic alterations in the *CDH1* gene, disrupts cellular cohesion and contributes to its characteristic infiltrative growth pattern. Despite its association with favorable prognostic markers, such as low Ki-67 proliferation index, positive estrogen receptor (ER) expression, and the absence of human epidermal growth factor receptor 2 (HER2) overexpression or amplification, evidence suggests that ILC may have worse long-term outcomes than stage-matched IDC [5].

While bones, liver, lungs, and brain are common metastatic sites for all breast cancers, ILC has an added propensity to metastasize to unusual sites such as the gastrointestinal (GI) tract, peritoneum, and adnexa [6]. Notably, ILC most commonly metastasizes to the stomach (4.5% of ILC versus 0.2% of IDC) [7,8]. Nevertheless, GI tract metastases overall remain uncommon, with a reported incidence of 4-18% in the stomach, 3-14% in the small bowel, 8% in the colon, and 2% in the rectum [9,10].

Here, we report a highly unusual case of metastatic ILC to gastric fundic gland polyps discovered during endoscopic evaluation for anemia, seven years after the initial diagnosis. While gastric metastasis from ILC has been reported, involvement limited to multiple fundic gland polyps without mucosal abnormalities is exceptionally rare. To our knowledge, no such case with this degree of polyp burden, especially in the absence of *APC*/*CTNNB1* mutations, has been previously reported.

## Case presentation

A 62-year-old postmenopausal female had a history of stage IIIC right-sided ILC of the breast, diagnosed five years earlier, when she noticed a lump in her right axilla. An initial mammogram revealed bilateral axillary lymph nodes, and follow-up ultrasound examinations ultimately led to a biopsy four months later, which confirmed metastatic ILC with extracapsular extension. Immunohistochemistry revealed ER/PR positivity and HER2/neu non-amplification. Further imaging, including magnetic resonance imaging (MRI) and positron emission tomography (PET)/computed tomography (CT), identified two non-mass-like enhancements in the right breast and abnormal right axillary uptake.

The patient began neoadjuvant chemotherapy with four cycles of dose-dense AC (Adriamycin (doxorubicin) and Cytoxan (cyclophosphamide)) followed by two cycles of dose-dense Taxol. Follow-up MRI showed excellent clinical response, with resolution of the non-mass-like enhancement and a reduction in the size of the right axillary lymph nodes. She subsequently underwent bilateral mastectomies, which showed multifocal residual ILC in the right breast, with the largest focus measuring 1.1 cm, and lobular carcinoma in situ. In total, 16 of 19 lymph nodes were positive, with the largest nodal metastasis measuring 1.6 cm and showing extracapsular extension. The final pathology stage was ypT1cN3aM0. The left breast showed atypical lobular hyperplasia but no evidence of carcinoma.

About seven months after the initial diagnosis, she completed adjuvant radiation therapy, receiving a total dose of 6,040 cGy to the right chest wall, supraclavicular fossa, and internal mammary nodes. She was also started on adjuvant hormonal therapy with Letrozole. Her tumor was classified as RCB-3 by Symmans’ criteria, indicating a 40% recurrence risk over five years. Her course over several years was complicated by iron deficiency anemia, with a hemoglobin reaching a level of 7.4 g/dL (normal: 12.0-15.5 g/dL). An upper GI endoscopy seven years after the initial presentation revealed over 50 erythematous and friable gastric polyps, which were thought to be the source of significant blood loss. The polyps, ranging in size from 3 mm to 20 mm, were primarily located in the cardia, gastric fundus, and body. Larger representative polyps were removed, and the remainder of the stomach and duodenum appeared normal. The patient also required a blood transfusion.

**Figure 1:**
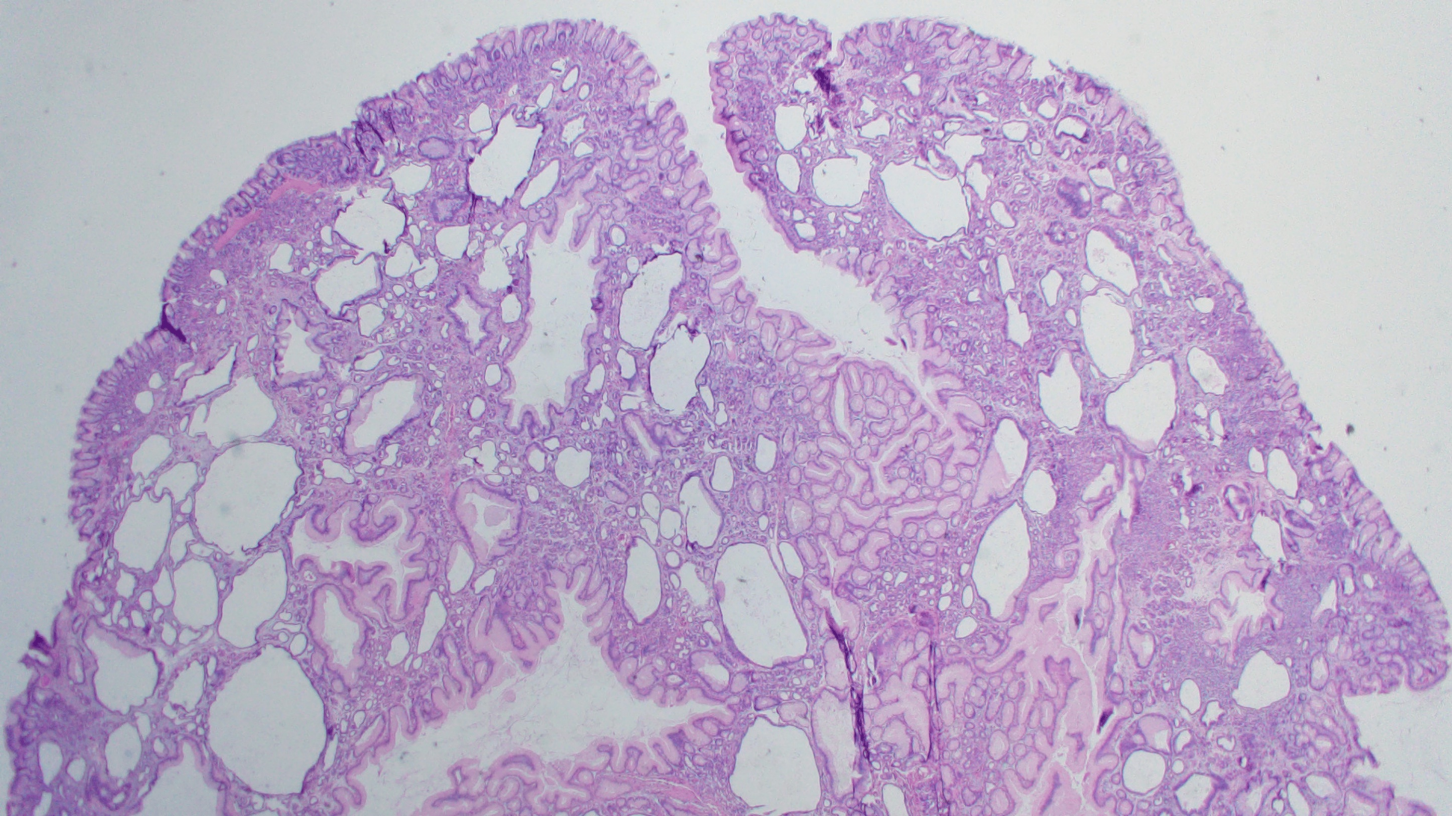
Low-power view of a gastric fundic gland polyp, showing cystically dilated glands within a loose stromal background.

**Figure 2:**
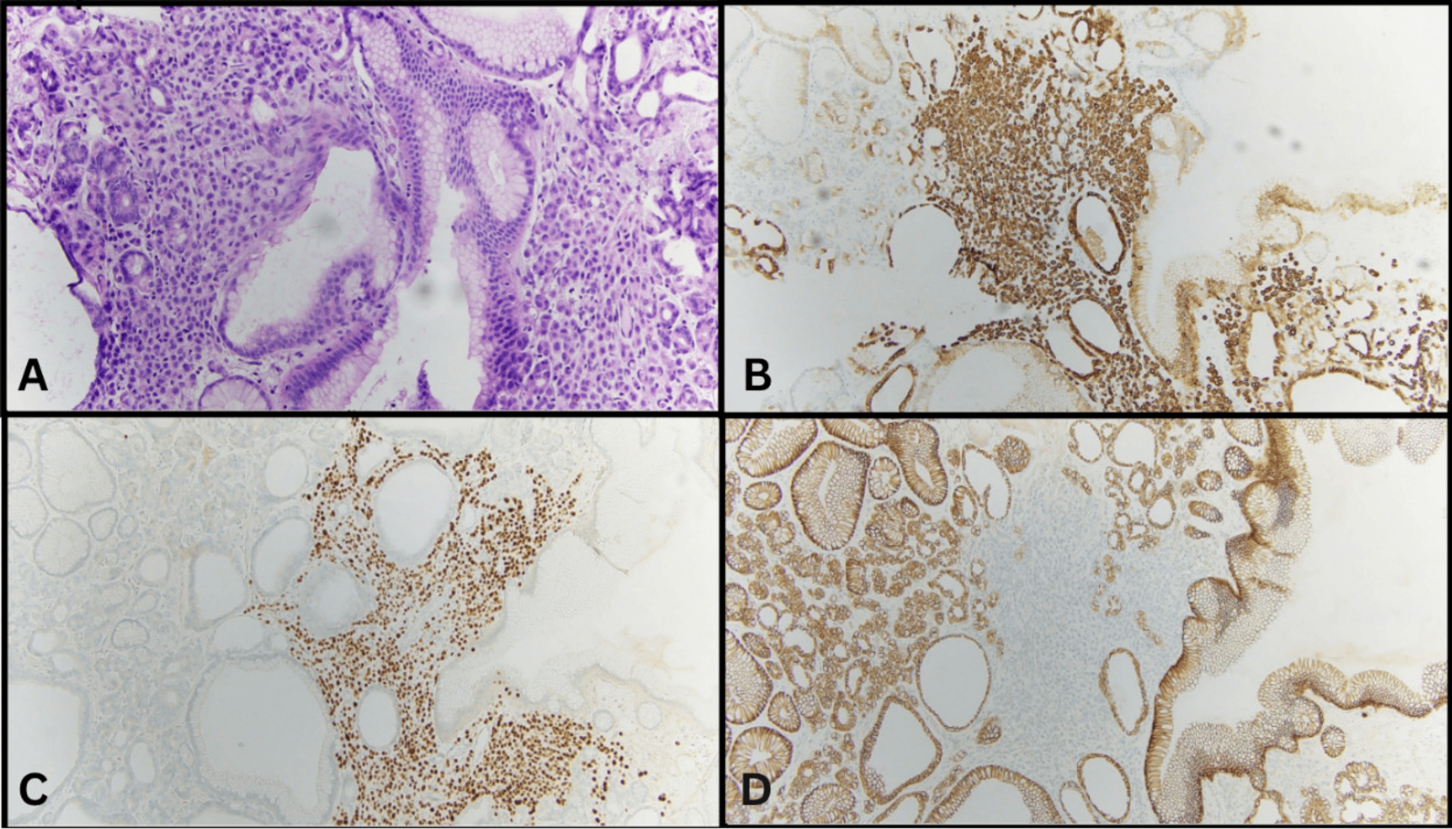
(A) Hematoxylin and eosin staining shows dyscohesive tumor cells within the gastric fundic gland polyp. (B) Immunohistochemical stain for cytokeratin 7 demonstrates diffuse positivity in the neoplastic cells. (C) GATA3 highlights the tumor cells, supporting a breast origin. (D) E-cadherin staining shows loss of expression in the tumor cells, a characteristic feature of invasive lobular carcinoma.

A total of 10 polyps, ranging from 0.6 cm to 2.0 cm, were received in the pathology laboratory. Histologic examination revealed fragments of gastric mucosa with cystically dilated oxyntic-type glands lined by mucous foveolar cells, parietal cells, and chief cells without evidence of cytologic dysplasia (Figure 1). In the lamina propria of eight polyps, there was a proliferation of epithelioid cells arranged in solid sheets and single files, some with signet ring morphology (Figure 2). These clusters ranged from 160 µm to 1,800 µm in size. By immunohistochemistry, these epithelioid cells were positive for CK7, ER (90%, strong), and GATA3 with loss of E-cadherin and negative for CK20 and CDX2, leading to a diagnosis of metastatic ILC to gastric fundic polyps (Figure 2). HER2/neu analysis by fluorescence in situ hybridization showed no amplification.

**Figure 3:**
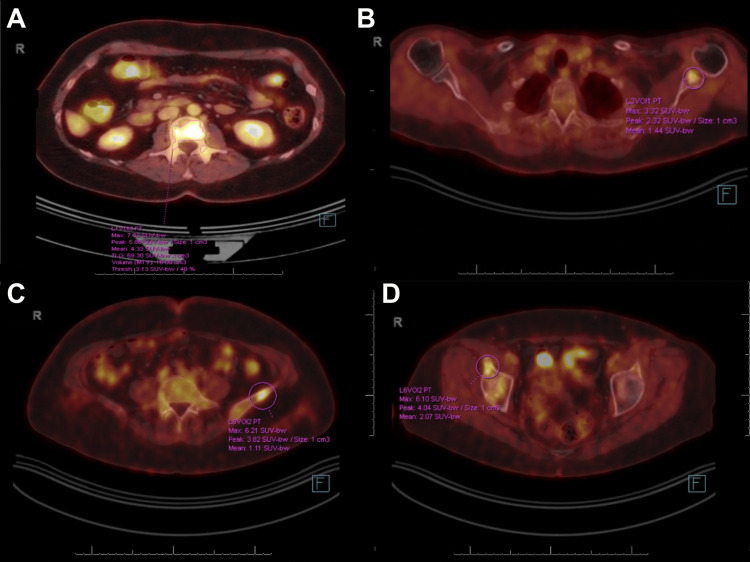
Axial fluorodeoxyglucose positron emission tomography/computed tomography images demonstrating metabolically active sclerotic osseous lesions in a patient with metastatic breast cancer. Focal hypermetabolic activity is seen in the L2 vertebral body (A, maximum standardized uptake value (SUVmax): 7.82), left glenoid (B, SUVmax: 3.32), left iliac bone (C, SUVmax: 6.21), and right iliac bone anterior to the hip (D, SUVmax: 6.10). These findings are consistent with osseous metastatic disease.

She received numerous adjuvant therapies, including fulvestrant, palbociclib, abemaciclib, and capecitabine. Subsequent scans, unfortunately, revealed widespread bone metastases to the ribs, appendicular and axial skeleton, as well as mesenteric and mediastinal lymphadenopathies. During this time, due to a strong family history of cancer, including her mother (pancreatic cancer), father (carcinoid tumor), brother (kidney cancer), and sister (melanoma), the patient underwent genetic counseling and additional testing, none of which revealed actionable mutations. Follow-up PET scan later showed a modest progression of bony metastases (Figure 3). The patient opted to discontinue cancer treatment and pursue the best supportive care. She passed away a few months later.

## Discussion

While gastric metastases from ILC have been previously reported, this case highlights a unique presentation involving over 50 fundic gland polyps, with 8/10 sampled polyps showing metastatic involvement. No mucosal abnormalities were observed endoscopically, and random gastric biopsies were not performed. It emphasizes the importance of keeping a high index of suspicion for disease recurrence/metastasis in this patient population. While ILC is recognized for its unique metastatic behavior, including a predilection for GI involvement, its presence within fundic gland polyps is an unusual finding that has not been widely reported. It also raises the question of metastasis as a likely trigger for these polyps.

Fundic gland polyps are the most frequently encountered gastric polyps, often identified incidentally during endoscopy as they are typically asymptomatic. They represent 50-77% of all gastric polyps and are found in up to 1.9% of the population [11,12]. Histologically, they are composed of cystically dilated fundic glands lined by normal foveolar epithelium and are generally regarded as non-neoplastic lesions, categorized as retention cysts or hamartomas. Fundic gland polyps may be sporadic, often associated with proton pump inhibitor use, or syndromic, in the context of familial adenomatous polyposis or gastric adenocarcinoma and proximal polyposis of the stomach syndromes [13]. Sporadic fundic gland polyps are associated with activating mutations in the *CTNNB1* gene encodingβ-catenin, while syndrome-associated fundic gland polyps are linked to germline mutations in the *APC* gene [14]. Of interest, in our patient, next-generation sequencing testing revealed no mutations in *CTNNB1* or *APC*, suggesting a distinct pathogenesis unrelated to these typical genetic alterations.

Interestingly, this patient had over 50 fundic gland polyps, with 8 of the 10 biopsied polyps harboring metastatic ILC. The exact relationship between fundic gland polyps and metastatic involvement remains unclear. Although most fundic gland polyps are not associated with metastasis, the absence of typical genetic alterations in our patient raises the possibility that these polyps developed as a reactive or hamartomatous response to infiltrating malignancy. Alternatively, fundic gland polyps could have provided additionally conducive vascularity or other microenvironment factors to attract and hone these metastasizing cells. Alternatively, the finding may reflect diffuse metastatic spread with incidental involvement of polyps, as the status of the non-polypoid gastric mucosa is unknown. Despite this limitation, the exclusive involvement of numerous benign-appearing polyps raises the possibility of selective polypoid tropism or a yet-unrecognized microenvironment favorable to metastatic seeding. Further investigation is warranted to determine whether specific features of fundic gland polyps, such as their stromal architecture or altered epithelial milieu, may predispose them to harbor metastatic disease.

Gastric metastases from breast cancer are recognized but still uncommon, with an estimated incidence ranging from 2.8% to 27% among patients with metastatic disease [15]. ILC, in particular, has a predilection for metastasizing to the GI tract, peritoneum, and ovaries, likely due to its histological characteristics and molecular profile resulting from loss of E-cadherin-modulated cell adhesion. The non-cohesive single-file infiltration pattern of ILC, driven by the loss of E-cadherin expression caused by *CDH1* gene alterations, may contribute to its capacity to invade and metastasize to sites with welcoming stromal microenvironments, such as the stomach.

Clinically, gastric metastases from breast cancer often mimic primary gastric malignancies, with nonspecific symptoms such as nausea, vomiting, dyspepsia, early satiety, epigastric pain, weight loss, and other signs [15,16]. In this case, anemia was the only presenting sign, highlighting the subtlety with which metastatic ILC can manifest. Endoscopically, gastric metastases may present as polyps, ulcers, or diffuse thickening resembling linitis plastica. These findings emphasize the importance of maintaining a high index of suspicion when evaluating gastric lesions, especially in patients with a history of breast cancer, even several years after the initial diagnosis and treatment.

Differentiating metastatic ILC from primary gastric carcinoma can be challenging due to overlapping histologic features, such as signet ring-shaped cells. Immunohistochemical analysis is crucial for accurate diagnosis [17]. In this case, the lesions in the fundic gland polyps were positive for CK7, ER, and GATA3 and negative for CK20 and CDX2, supporting metastatic ILC and ruling out primary gastric carcinoma.

The management of gastric metastases from breast cancer is primarily systemic, with a focus on hormone therapy or chemotherapy tailored to the molecular profile of the tumor. Surgical intervention is typically reserved for palliative purposes, such as controlling bleeding or obstruction, or for solitary, resectable GI metastases [17]. In contrast, the treatment of primary gastric malignancies is centered on surgical resection in the absence of distant metastases. In this case, the patient underwent multiple lines of systemic therapy with limited response, underscoring the challenges of treating advanced metastatic disease.

## Conclusions

This case is particularly unique as it describes metastatic ILC to a commonly encountered gastric polyp that is almost always benign. Despite having more than 50 fundic gland polyps endoscopically, no underlying genetic abnormality was documented to provide a usual explanation for fundic gland polyps in this patient. Metastases of ILC after seven years to 8/10 sampled fundic gland polyps would, therefore, suggest more than a subtle coincidence and raise an interest in exploring metastasis as another cause, or mitigating factor, for developing these otherwise very common polyps. Being very common and almost always benign, fundic gland polyps are often easily overlooked, and therefore, this case highlights the importance of added scrutiny in patients with a history of malignancy, especially ILC. To our knowledge, this pattern of metastatic involvement within fundic gland polyps has been rarely reported in the literature, highlighting the need for increased vigilance when evaluating such lesions in patients with a history of lobular breast carcinoma. Although uncommon, this case underscores the importance of including metastatic disease in the differential diagnosis of gastric findings, even years after the initial diagnosis and treatment of ILC.
